# Y-27632 Impairs Angiogenesis on Extra-Embryonic Vasculature in Post-Gastrulation Chick Embryos

**DOI:** 10.3390/toxics11020134

**Published:** 2023-01-30

**Authors:** Johannes W. Duess, Jan-Hendrik Gosemann, Anna Kaskova Gheorghescu, Prem Puri, Jennifer Thompson

**Affiliations:** 1 Department of Pediatric Surgery, University of Leipzig, 04103 Leipzig, Germany; 2 National Children’s Research Centre, Our Lady’s Children’s Hospital, Crumlin, 12 Dublin, Ireland; 3 School of Medicine and Medical Science, University College Dublin, Belfield, 4 Dublin, Ireland

**Keywords:** Y-27632, ROCK, post-gastrulation, chick embryo, angiogenesis, ventral body wall defect

## Abstract

Y-27632 inhibits Rho-associated coiled-coil-containing protein kinase (ROCK) signaling, which is involved in various embryonic developmental processes, including angiogenesis, by controlling actin cytoskeleton assembly and cell contractility. Administration of Y-27632 impairs cytoskeletal arrangements in post-gastrulation chick embryos, leading to ventral body wall defects (VBWDs). Impaired angiogenesis has been hypothesized to contribute to VBWDs. ROCK is essential in transmitting signals downstream of vascular endothelial growth factor (VEGF). VEGF-mediated angiogenesis induces gene expressions and alterations of the actin cytoskeleton upon binding to VEGF receptors (VEGFRs). The aim of this study was to investigate effects of Y-27632 on angiogenesis in post-gastrulation chick embryos during early embryogenesis. After 60 h incubation, embryos in shell-less culture were treated with Y-27632 or vehicle for controls. Y-27632-treated embryos showed reduced extra-embryonic blood vessel formation with impaired circulation of the yolk sac, confirmed by fractal analysis. Western blot confirmed impaired ROCK downstream signaling by decreased expression of phosphorylated myosin light chain. Interestingly, RT-PCR demonstrated increased gene expression of VEGF and VEGFR-2 1 h post-treatment. Protein levels of VEGF were higher in Y-27632-treated embryos at 8 h following treatment, whereas no difference was seen in membranes. We hypothesize that administration of Y-27632 impairs vessel formation during angiogenesis, which may contribute to failure of VWB closure, causing VBWDs.

## 1. Introduction

Ventral body wall defects (VBWDs) are human birth anomalies reportedly present in about one in two thousand live births, with omphalocele and gastroschisis being the most frequently observed defects [1,2]. Although similar in that both conditions involve herniation of abdominal contents through the ventral body wall, they differ in that omphalocele occurs through the base of the umbilical cord and has a peritoneal covering, whereas gastroschisis occurs usually to the right of the umbilical cord and herniated organs have no peritoneal covering. Both conditions are relatively infrequent, and their exact causes are still unknown [3,4].

Omphalocele may occur as an isolated lesion but may also occur as part of various syndromes associated with chromosomal abnormalities, such as Beckwith–Wiedmann, Down’s, and Patau syndromes. The VBWD in omphalocele is thought to result from failure of the midgut loop to rotate and return to the abdominal cavity from the physiological hernia of early pregnancy. Incomplete development and migration of the lateral plate mesoderm to form the body wall and incomplete differentiation and migration of somitic myotomes to form precursors of the body wall musculature may also contribute. Gene mutations and environmental factors have also been implicated in omphalocele formation [3,5,6,7].

The etiology of gastroschisis is reported to be the result of a disruption to blood flow to the developing body wall, leading to deficient development of the affected tissues and subsequently to an intestinal herniation through the defect [3,8]. It has been hypothesized that the abnormal involution of the right umbilical vein in particular leads to impaired growth and viability of the surrounding mesenchyme, which then results in a defect of skin formation in the periumbilical area and subsequent rupture of the intestines through the defect [1,7,9,10]. In addition to the latter, Hoyme stated that disruption of the omphalomesenteric artery leads to infarction and necrosis at the base of the umbilical cord with subsequent gut herniation [1,11]. The importance of the appropriate progress of angiogenic activities during embryogenesis is highlighted in studies on vasoactive substances. Mothers who took pseudoephedrines and/or analgesics (such as aspirin) during the first trimester of pregnancy were more likely to have a baby that had gastroschisis [12]. Smoking cigarettes during pregnancy is also linked with VBWDs during embryogenesis [13,14]. In addition, alcohol consumption and recreational drug use, particularly cocaine, has been correlated with a higher incidence of gastroschisis [15].

Since a direct correlation between an environmental factor and body wall malformation is difficult to verify, animal models have been used to investigate hypotheses on the relationship between VBWDs and angiogenesis. In chick embryos, the administration of the heavy metal cadmium (Cd) following completion of gastrulation at Hamburger–Hamilton (H-H) stages 16–17 altered the extra-embryonic vascular branching pattern, leading to an isolated morphological defect, similar to human VBWDs [10,16,17,18]. Rho-associated coiled-coil-containing protein kinase (ROCK) gene expression levels were found to be significantly decreased in chick embryos following Cd exposure [19]. In other studies, chick embryos exposed at H-H stages (16–17) to the pyridine derivative Y-27632 formed abnormal somites and kinking in the lumbosacral region, findings similar to those observed following Cd exposure. Y-27632 inhibits ROCK signaling, which is involved in numerous developmental processes during embryogenesis, such as cell adhesion, motility, proliferation, differentiation, and apoptosis, primarily by controlling actin cytoskeleton assembly and cell contractility. Western blot analysis implicated interference with ROCK downstream signaling in affected embryos. In addition, administration of Y-27632 at H-H stages 16–17 has also been shown to lead to increased cell death in somites, the neural tube, and ectoderm, as well as to alteration in cytoskeletal arrangements during early chick embryogenesis, all of which may contribute to failure of ventral body wall closure at later stages of development in chick embryos [20,21,22].

Angiogenesis, also known as neovascularization, is the process of new capillary formation from pre-existing vessels through endothelial cell proliferation and stem cell recruitment in combination with morphogenesis [23,24,25]. It must be distinguished from vasculogenesis, a process whereby vessels are formed de novo from endothelial cell precursors termed angioblasts and which occurs in the extra- and intra-embryonic tissues of embryos [26,27,28,29]. Angioblasts and newly formed endothelial cells proliferate, migrate, and remodel into tubular structures and fuse into larger vessels to create an interconnecting network of vessels referred to as the primitive capillary plexus [26,30]. With subsequent growth, this primitive vascular plexus remodels during the process of angiogenesis [31].

Vascular endothelial growth factor (VEGF) is one of the most important factors regulating angiogenesis during embryogenesis, including vascular endothelial cell migration, proliferation, and permeability, as well as skeletal growth and reproductive functions [32]. The biological effects of VEGF are mediated through three structurally related VEGF receptor tyrosine kinases (VEGFR), namely VEGFR-1, VEGFR-2, and VEGFR-3 [32,33,34]. VEGFR-2, also designated as KDR or Flk1, is the predominant receptor in angiogenic signaling and the main VEGF receptor on endothelial cells. It is essential for the regulation of endothelial cell migration, proliferation, differentiation, and survival as well as vessel permeability and dilatation [33,35]. VEGF induces hyperpermeability by enhancing nitric oxide (NO) production, which promotes endothelial NO synthase (eNOS) and inducible NOS (iNOS) expression in endothelial cells [27]. In addition, VEGFR-2 expression levels are particularly increased during embryonic vasculogenesis and angiogenesis [33].

Although the importance of VEGF signaling during angiogenesis is well established, the precise morphogenetic processes involved in the organization of proliferating endothelial cells into new blood vessels are still unknown. It has been hypothesized that major mechanisms are controlled through cytoskeletal elements [23]. Therefore, the role of members of the Rho family of small GTPases as key regulators of angiogenesis has been investigated, as they modulate the extra-cellular matrix and regulate migration, proliferation, morphogenesis, and survival through the cytoskeleton. RhoA signaling and its downstream target Rho-associated coiled-coil-containing protein kinase (ROCK) have been shown to play an important role in VEGF-dependent in vivo angiogenesis and in initial steps of in vitro endothelial cord assembly [23,36,37,38,39,40,41]. ROCK proteins phosphorylate various substrates, including myosin light chain (MLC) phosphatase and LIM-kinases (LIMK-1, LIMK-2) and activate them to phosphorylate and inactivate cofilin-1 (also known as non-muscle cofilin) or cofilin-2 (also known as muscle cofilin), which leads to inhibition of actin depolymerization and collectively to a reorganization of the actin cytoskeleton [42,43]. It was supposed that Rho proteins are downstream targets of VEGF-mediated signaling, leading to the activation of Rho GTPases after VEGF and VEGFR-2 stimulation, therefore controlling endothelial cell migration [30,36,44,45]. However, several studies have identified a critical and selective role for RhoA in the regulation of endothelial cell assembly into new blood vessels [23].

The research goal of the current study is largely mechanistic in terms of the effect of Y-27632 on angiogenesis, focusing on the extra-embryonic vascular branching pattern in chick embryos following exposure to teratogenic doses of this drug after 60 h incubation [21]. Moreover, the impact of Y-27632 on VEGF signaling during angiogenesis and different genes involved in vessel formation are investigated. The incubation time of 60 h is a point in chick development at which gastrulation is completing and body wall folding is initiating. Embryos were explanted using the Dugan method [46], which permits staging prior to treatment and intermittent review of the extra-embryonic vasculature and developmental progress prior to harvesting embryos.

## 2. Materials and Methods

Fertilized unincubated eggs of the Ross strain were obtained from a commercial hatchery (Enfield Broiler Breeders Ltd., Maynooth, Co. Kildare, Ireland). They were incubated at 38 °C in a forced draught incubator (Shell Lab) at 65–75% humidity until they reached the required stage for treatment. All animal studies were carried out in accordance with the established protocols of the institute. After 60 h of incubation, chick embryos that had attained H-H stages 16–17 (distinguished by the presence of the cervical flexure, absent or rudimentary limb buds, blood islands below the tail region, and otherwise well-developed extra-embryonic vasculature) were explanted into shell-less culture using a method adapted from Dugan et al. [46].

This method for ex ovo culture of chick embryos involves the explantation of the embryo and egg contents into a concave well made with cling film, which is attached to the exterior of a polystyrene cup using an elastic band, ballasted with 30–50 mL of water. The eggs were removed from the incubator and left for 5–10 min at room temperature, allowing them to cool down and thus reducing the possibility of yolk rupture upon explantation. Subsequently, the shells were cracked, and the contents were emptied into the wells with the blastodisc facing upwards. Embryos were staged according to H-H parameters prior to treatment. Using an adjustable volume pipette, 50 µL of 500 µM of ROCK inhibitor Y-27632 (Y-27632 dihydrochloride, Sigma-Aldrich, cat.# Y0503, Ireland), diluted in chick saline (0.72 g NaCl/100 mL dH_2_O), was applied to the center of the blastodisc, and entered the embryo and vitelline membranes by a process of diffusion. Embryos in the control group received 50 µL of vehicle. The dose of 500 µM of Y-27632 has recently been shown to result in the optimal balance between mortality and morphological abnormality rate [21].

Embryos were assessed 8 h, 24 h, 48 h, and 4 d after treatment at 60 h for development of extra-embryonic vasculature. Measurements, including length of sinus terminalis (ST), size of area vasculosa (AV), and absolute area vasculosa (AOV), as well as vascular index (VI), were performed at 60 + 24 h to analyze differences in formation of extra-embryonic membrane (EEM) vasculature between controls and Y-27632-treated embryos. Fractal dimensions were calculated to study the branch morphological pattern of right- and left-sided extra-embryonic vessels (D-right, D-left) at 4 d following treatment. Western blot was performed at 8 h post-treatment to determine whether there was interference with ROCK signaling. Relative gene expression levels of pro- and anti-angiogenic factors were analyzed 1 h, 4 h, and 8 h (for membranes) or 24 h (for embryos) after treatment at 60 h. Enzyme-Linked Immunosorbent Assays (ELISA) for VEGF and VEGFR-2 were performed at 8 h and 24 h following treatment. These time points are summarized in Table 1.

Embryos and EEM were harvested and assessed for stage development and anomalies using the dissection microscope (Leica S6E). Dead embryos were recorded and discarded. Surviving chicks were photographed with a single-lens reflex camera to study the formation of the extra-embryonic vasculature or with the stereomicroscope (Olympus SZX12) attached to a camera port (Olympus DP70) to analyze the morphological development of chick embryos.

### 2.1. Measurements of EEM Formation

**Figure 1:**
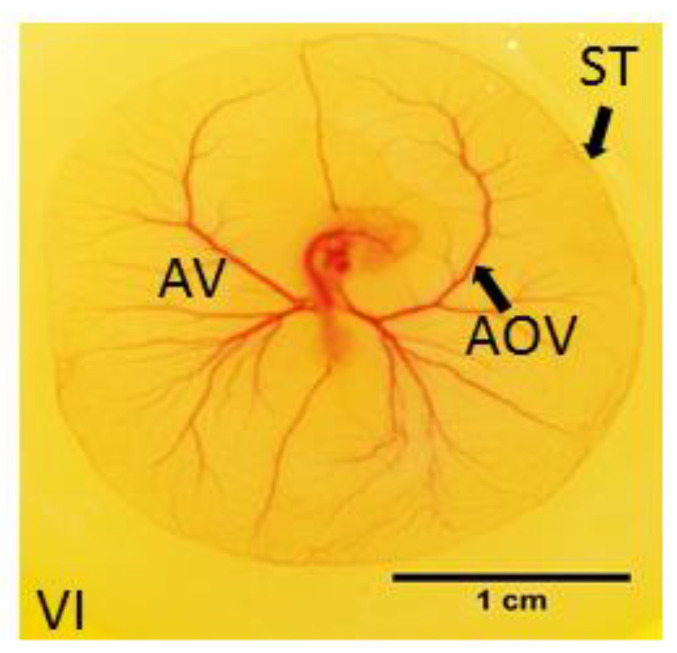
Parameters for measurement of extra-embryonic vasculature formation: sinus terminalis (ST, in cm), size of area vasculosa (AV, in cm^2^), absolute area vasculosa (AOV, in cm^2^), and vascular index (VI, in %). Scale bar 1 cm.

Eight chick embryos were randomly collected from the control and experimental group 24 h following treatment at 60 h to measure parameters of the extra-embryonic vasculature (Figure 1). The length of ST (in cm) and the size of the area vasculosa (AV, in cm^2^) were determined using Fiji^®^ image processing software. The size of AV was calculated by subtracting the chick embryo expanse from the area surrounded by the ST. The area occupied by vessels of the AV (AOV in cm^2^) was measured using Adobe Photoshop (Adobe Systems). VI (in %) is expressed as the percentage of AOV from AV. Measurements were performed double-blinded by two independent investigators.

### 2.2. Fractal Analysis

Eight chick embryos were randomly collected from the control and experimental groups 4 d following treatment at 60 h to calculate fractal dimensions for the study of the branch morphological pattern of right- and left-sided extra-embryonic vessels (D_f_-right, D_f_-left). Fractal dimensions (D_f_) were determined using the “box counting” method in Fiji^®^ image processing software. An area covering extra-embryonic vessels on one side was randomly selected at 1, 3, 5, 7, 9, and 11 o’clock and converted to an 8-bit image. To analyze the pattern branching, the images were converted to dichotomic binarized (black and white) pictures and skeletonized to obtain a final picture where each vessel had the structural shape of the object, being in this case a direct representation of total vessel length [47]. D_f_ was then calculated from the skeletonized line tracing using the box counting method [47,48,49]. The logarithm of the number of boxes containing a segment is plotted against the logarithm of the number of boxes covering the image, and the D_f_ is calculated from the slope by the computer program [50]. Fractal analysis is thought to be more sensitive to changes in vascular patterns when skeletons rather than segmented images are considered [47,50]. Measurements were performed double-blinded by two independent investigators.

### 2.3. Western Blot

Western blot (WB) was performed in triplicate to confirm inhibition of ROCK downstream signaling in embryos and EEM at the teratogenic dose of 500 µM of Y-27632.

Following treatment with the aforementioned dose of Y-27632 or vehicle, chick embryos were harvested and sonicated in 200 µL RIPA (Radio Immuno Precipitation Assay) buffer. Protein concentrations were then measured using Bradford assays and diluted with gel loading buffer Laemmli (Sigma-Aldrich, Arklow, Co. Wicklow, Ireland) prior to gel loading. Proteins were separated electrophoretically in 10% SDS-PAGE and transferred to a 0.45 µm nitrocellulose membrane by Western blotting for 90 min at 30 V. Following blotting, the membranes were blocked in 3% BSA-0.05% Tween for 30 min to overnight. Primary antibody (Anti-Myosin light chain (phospho S20), 1:500, Abcam, Cambridge, UK) incubation was performed from 90 min to overnight. After extensive washing (four times in PBS-0.05% Tween 20 or 4 h for overnight incubation) the membranes were incubated with the secondary antibody (Anti-rabbit IgG, HRP-linked Antibody, 1:5000, Cell Signaling Technology-Isis Ltd., Stillorgan, Co. Dublin, Ireland) for 90 min followed by extensive washing. Protein detection was performed with PIERCE chemiluminescence kit (ECL Western blotting substrate Thermo Scientific Pierce, Fisher Scientific, Dublin, Ireland). Beta-actin (Anti-beta Actin antibody, Abcam, Cambridge, UK) was used to control for equal loading and transfer of the samples. Bands of WB were quantified using densitometry.

### 2.4. Real-Time Quantitative Reverse Transcription Polymerase Chain Reaction (RT-PCR)

RT-PCR was performed to evaluate the relative mRNA levels of VEGF, VEGFR-2, Ang-2, and iNOS in embryos and EEM harvested 1 h, 4 h, and 8 h (for membranes) or 24 h (for embryos) after treatment in shell-less culture. Total RNA of twelve embryos (six controls, six Y-27632-treated embryos) at each time point was isolated using TRIzol^®^ reagent (Bio-Sciences, cat.# 15596-026, Dublin, Ireland) according to the recommended protocol. For reverse transcription (RT), first strand cDNA was synthesized from RNA by using a Transcriptor High Fidelity cDNA Synthesis Kit (Roche, cat.# 05091284001, UK). Following RT at 44° for 60 min, polymerase chain reaction (PCR) was performed using a LightCycler^®^ 480 SYBR Green I Master (Roche, cat.# 04887352001, West Sussex, UK). After an initial denaturation step of 5 min at 95°, PCR cycles of amplification for target primers were carried out. Relative levels of gene expression were measured using a LightCycler 480 (Roche, UK). The relative mRNA expression levels of target genes were normalized against the level of GAPDH gene expression in each sample. Experiments were carried out in triplicate for each data point. The specific primer pairs used in this study are listed in Table 2.

### 2.5. Enzyme-Linked Immunosorbent Assay (ELISA)

In embryos and EEM harvested at 8 h and 24 h following treatment, ELISA was performed in 16 embryos and membranes (eight from each group for one time point) using ELISA kits for VEGF-A and VEGFR-2 (Chicken vascular endothelial growth factor A ELISA Kit, MyBioSource, Inc., USA; Chicken VEGFR-2/KDR ELISA Kit, MyBioSource, Inc., San Diego, CA, USA) according to the manufacturer’s protocol. Experiments were carried out in technical duplicate for each data point. The results were measured at 450 nm using the Synerg Mx microplate reader (BioTek, Winooski, VT, USA) immediately after adding the stop solution.

### 2.6. Statistical Analysis

All numerical data are shown as number of cases (%) or mean (± standard deviation, SD). Differences in extra-embryonic measurements, fractal dimension, densitometry, and relative mRNA and protein expressions were compared using 2-tailed unpaired *t* test when data were normally distributed or Mann–Whitney *U* test when data deviated from normal distribution. A *p*-value < 0.05 was considered statistically significant.

## 3. Results

### 3.1. Effects of Y-27632 on Angiogenic Development

**Figure 2:**
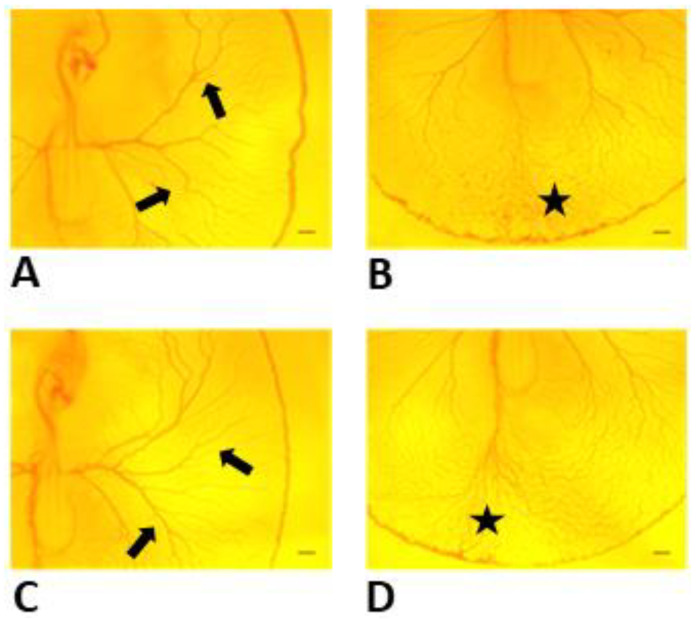
Development of extra-embryonic vasculature of chick embryos prior to treatment at 60 h incubation. Well-developed extra-embryonic vasculature (**A**,**C**; arrows) and blood islands in the tail region (**B**,**D**; asterisks) were evident prior to treatment. Control embryos were treated with vehicle (**A**,**B**), and embryos in the experimental group (**C**,**D**) received 500 µM of Y-27632. Scale bars 200 µm.

At 60 h incubation, embryos in both groups showed blood islands in the tail region and well-developed extra-embryonic vasculature (Figure 2A–D).

**Figure 3:**
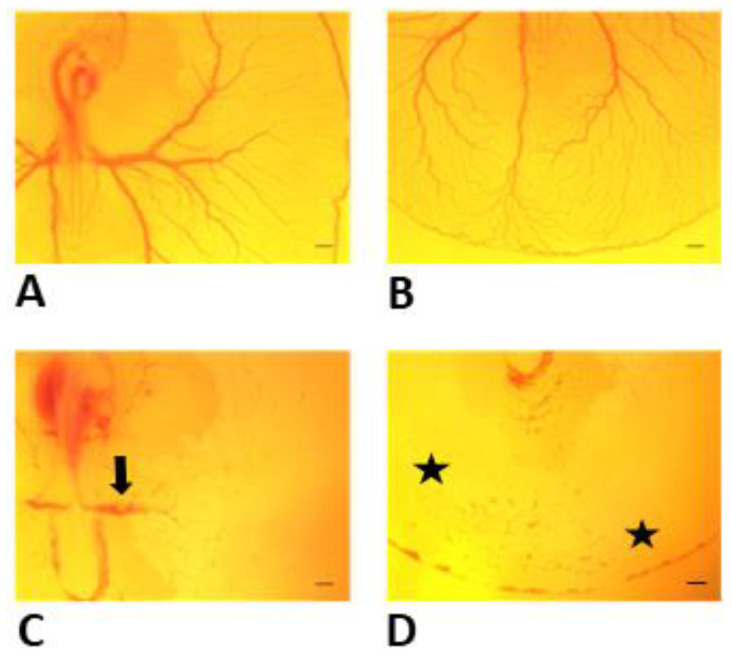
Development of extra-embryonic vasculature of control embryo (**A**,**B**) and Y-27632 (500 µM)-treated embryo (**C**,**D**) at 8 h following treatment at 60 h. In the experimental group, the majority of embryos revealed improper formation of omphalomesenteric vessels (**C**, arrow) and vitelline vessels in the tail region (**D**, asterisks) when compared with controls (**A**,**B**). Scale bars 200 µm.

At 8 h following treatment, all controls (*n* = 54) and 78 (97.5%) out of 80 Y-27632-treated embryos were alive. In the experimental group, 67 (85.8%) embryos demonstrated improper formation of extra-embryonic vasculature. Omphalomesenteric vessels appeared to be disrupted or strikingly underdeveloped, and immaturely formed blood islands were also evident in the tail region at 60 + 8 h in comparison to controls (Figure 3A,B). The thin vitelline vessels and pre-existing vascular architecture appeared severely impaired in the experimental group post-treatment (Figure 3C,D). Out of the 67 embryos with impaired blood vessel formation, 60 (89.6%) revealed morphological abnormalities, such as improperly developed somites in the tail region. A significant weight difference between controls and Y-27632-treated embryos with abnormalities in vessel formation and morphology was also found (0.0151 g ± 0.003 g vs. 0.0067 g ± 0.002 g, *p* < 0.001) at 60 + 8 h.

**Figure 4:**
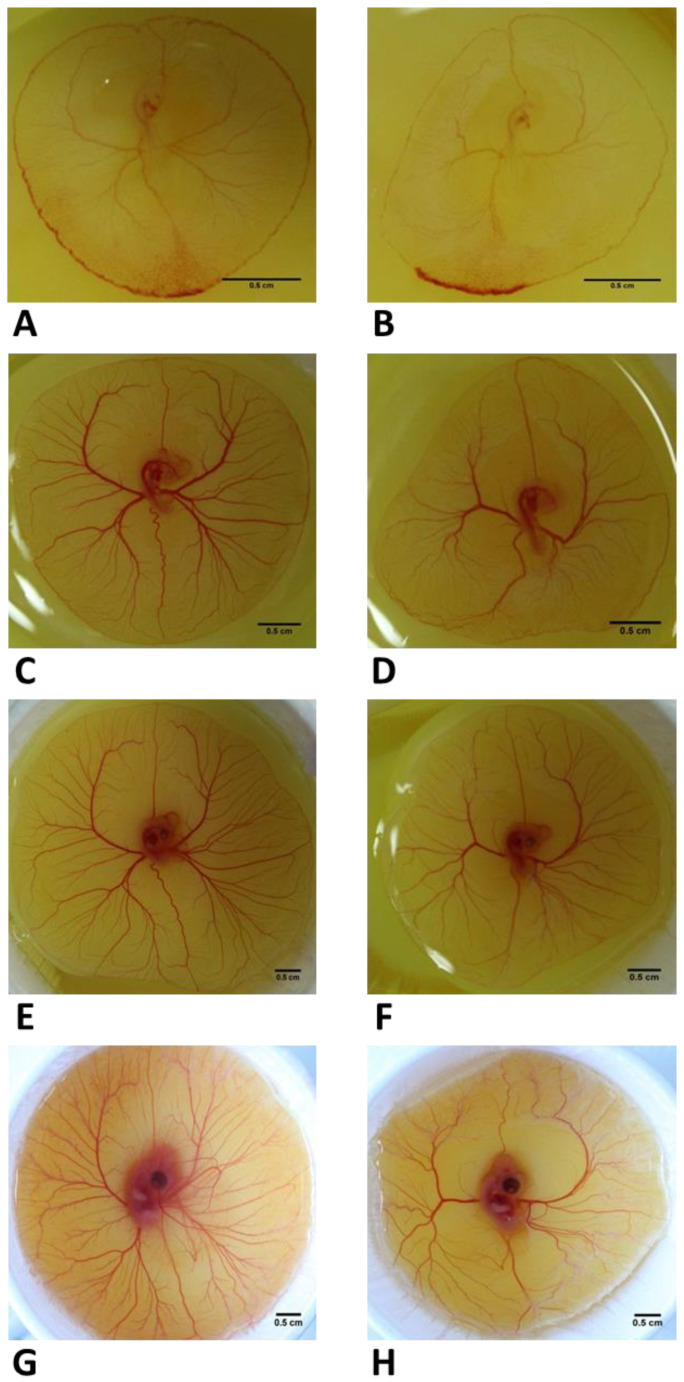
Development extra-embryonic vasculature of control embryo (**A**,**C**,**E**,**G**) and Y-27632 (500 µM)-treated embryo (**B**,**D**,**F**,**H**) at 60 h ((**A**,**B**)–prior to treatment), 60 + 24 h (**C**,**D**), 60 + 48 h (**E**,**F**), and 60 h + 4 d (**G**,**H**). Following administration of agents, embryos in the experimental group revealed strikingly impaired neovascularization with reduced formation of blood vessels and reduced circulation of the yolk sac when compared with controls at 24 h (**C**,**D**), 48 h (**E**,**F**), and 4 d (**G**,**H**) post-treatment. Scale bars 0.5 cm.

For the analysis of extra-embryonic angiogenesis at later stages of development, 38 embryos were treated with vehicle for the control group and 69 embryos with 500 µM of Y-27632 for the experimental group at 60 h incubation. Following 24 h, 48 h, and 4 d in the incubator, embryos were assessed for the formation of extra-embryonic vasculature at each time point. Prior to treatment, embryos in both groups demonstrated well-developed extra-embryonic vasculature and blood islands in the tail region (Figure 4A,B). After 24 h, 36 (94.7%) controls and 43 (62.3%) Y-27632-treated embryos were alive at examination. In controls, 33 (91.7%) embryos showed normal extra-embryonic vasculature. In contrast, 23 (53.5%) chick embryos in the experimental group showed reduced extra-embryonic blood vessel formation with impaired circulation of the yolk sac (Figure 4D,F,H) when compared with controls (Figure 4C,E,G) during the time period of investigation. Out of these 23 embryos, morphological abnormalities in the somites and the neural tube were detected in 21 (91.3%) embryos. A significant weight difference between controls and Y-27632-treated embryos with abnormalities in vessel formation and morphology was also found (0.0263 g ± 0.005 g vs. 0.0185 g ± 0.0052 g, *p* < 0.05) at 60 + 24 h.

### 3.2. Measurements of EEM Formation

**Figure 5:**
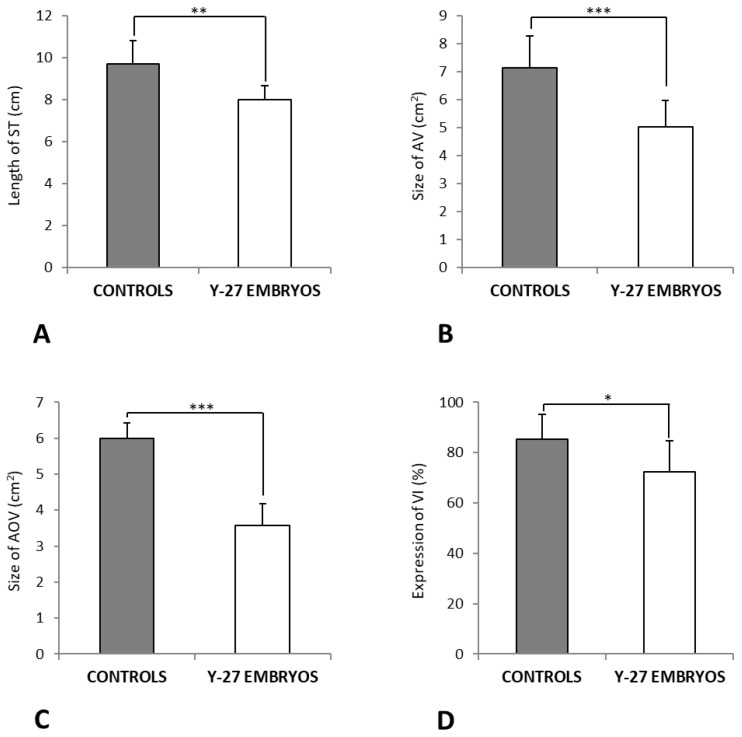
Measurements of EEM formation at 60 + 24 h. There was a significant difference in the mean length of ST (**A**, ** *p* = 0.001), mean size of AV (**B**, *** *p* < 0.001), mean size of AOV (**C**, *** *p* < 0.001), and mean VI (**D**, * *p* < 0.05) between controls and Y-27632 (500 µM)-treated embryos. The vertical bars represent the SD. *y-axis*: Length of ST in cm (**A**), Size of AV and AOV in cm^2^ (**B**,**C**), and Expression of VI in % (**D**).

The mean length of ST was 9.7 cm (±1.1) in controls vs. 8 cm (±0.6) in Y-27632-treated embryos (*p* < 0.05, Figure 5A). The mean size of AV was 7.1 cm² (± 1.1) vs. 5 cm² (±1.0) (*p* < 0.05, Figure 5B). There was also a significant difference in the mean AOV between the two groups (6 cm² (±0.4) vs. 3.6 cm² (±0.6), *p* < 0.05) (Figure 5C). The mean VI was 85.3% (±10.0) in controls compared to 72.5% (± 12.2) in Y-27632-treated embryos (*p* < 0.05, Figure 5D).

### 3.3. Fractal Analysis

**Figure 6:**
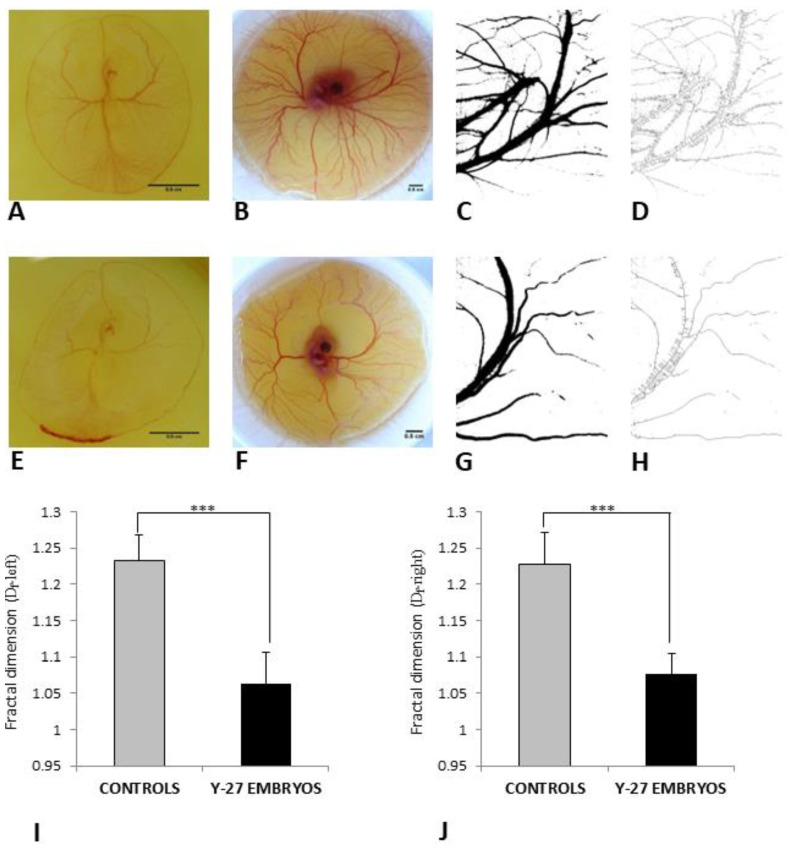
Fractal analysis of control embryo (**A**–**D**) and Y-27632 (500 µM)-treated embryo (**E**–**H**) with D_f_-left (**I**) and D_f_-right (**J**) at 60 h + 4 d. Prior to treatment, embryos in both groups showed blood islands in the tail region and well-developed extra-embryonic vasculature (**A**,**E**). Control embryos developed a more complex branching pattern (**B**–**D**) in comparison to embryos in the experimental group (**F**–**H**). D_f_-left was significantly lower in Y-27632 (500 µM)-treated embryos in comparison to controls (**I**, *** *p* < 0.001). There was also a significant difference in D_f_-right between controls and embryos in the experimental group (**J**, *** *p* < 0.001). The color image version (**B**,**F**). The segmented version (**C**,**G**). The skeletonized version (**D**,**H**). The vertical bars represent the SD. *y-axis*: Fractal dimension (D_f_-left, (**I**); D_f_-right, (**J**)). Scale bars: 0.5 cm.

At examination, controls embryos showed a more complex branching pattern from 60 h incubation (Figure 6A) to 4 d following treatment (Figure 6B–D) in comparison to a paucity of vessels in Y-27632-treated embryos (Figure 6E–H) during the time period of investigation. A significant difference in the mean D_f_-left was found between controls and Y-27632-treated embryos (1.232 ± 0.035 vs. 1.064 ± 0.043, *p* < 0.05) (Figure 6I). The mean D_f_-right was also significantly lower in control embryos compared with embryos in the experimental group (1.227 ± 0.044 vs. 1.077 ± 0.029, *p* < 0.05) (Figure 6J).

### 3.4. Western Blot

**Figure 7:**
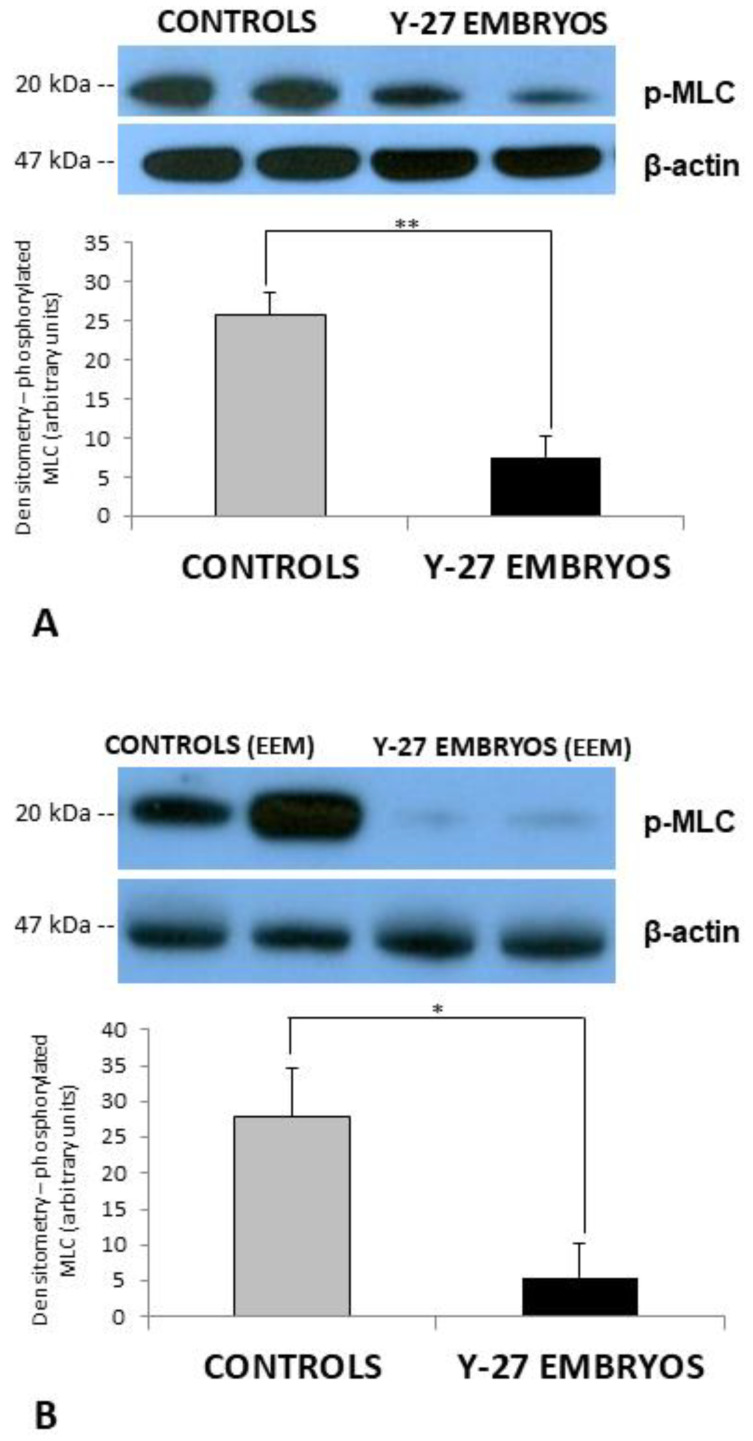
Embryos treated with vehicle (controls, **A**) or Y-27632 (500 µM, **A**) and EEM of controls (**B**) or Y-27632-treated embryos (**B**) were subjected to Western blot at 60 + 8 h to detect phosphorylated MLC. The results show that the amount of phosphorylated MLC was significantly reduced in Y-27632-treated embryos in comparison to controls (**A**, ** *p* < 0.001 and **B**, * *p* < 0.05). The vertical bars represent the SD. y-axis: Densitometry (grey levels, arbitrary units).

Western blot analysis confirmed Y-27632 interference with ROCK downstream signaling in embryos treated with the teratogenic dose of 500 µM of Y-27632, with significant reduction in the expression of phosphorylated MLC (p-MLC) at 8 h following treatment (*p* < 0.05, Figure 7A). In addition, ROCK signaling was also impaired in EEM of Y-27632-treated embryos, leading to a significant reduction in the expression of p-MLC at the same time point (*p* < 0.05, Figure 7B).

### 3.5. RT-PCR of VEGF and VEGFR-2

Effects of Y-27632 on VEGF and VEGFR-2 signaling were analyzed at 1 h, 4 h, and 8 h (for membranes) or 24 h (for embryos) following treatment at 60 h incubation.

**Figure 8:**
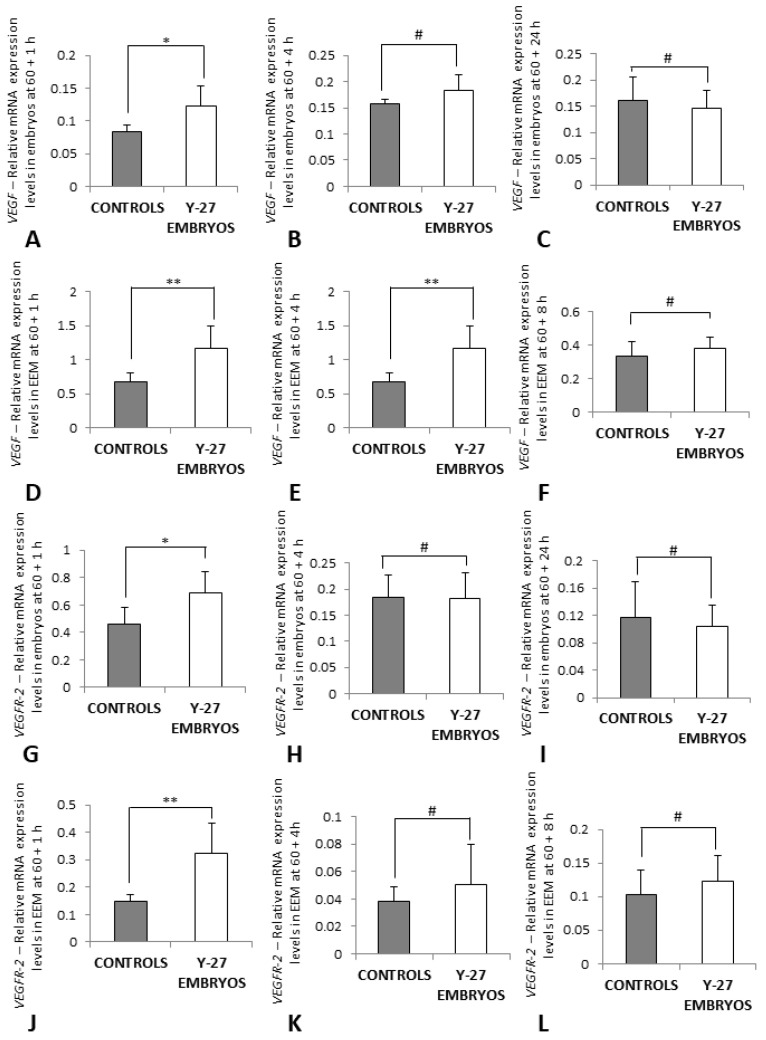
The relative mRNA expression levels of *VEGF* (**A**–**F**) and *VEGFR-2* (**G**–**L**) in controls and embryos treated with Y-27632 (500 µM) at 60 h incubation. Embryos in (**A**–**C**,**G**–**I**), EEM in (**D**,**F**,**J**–**L**). The expression levels of *VEGF* and *VEGF*R-2 at 60 + 1 h were significantly higher in the treatment group when compared to controls (**A**,**G**, * *p* < 0.05 and **D**,**J**, ** *p* = 0.009). The expression levels of *VEGF* and *VEGFR-2* at other time points (**B**,**C**,**E**,**F**,**H**,**I**,**K**,**L**) were not different between controls and Y-27632-treated embryos (# *p* > 0.05). The vertical bars represent the SD. *y*-axis: Relative mRNA Expression Levels.

In Y-27632-treated embryos, relative mRNA expression levels of *VEGF* were significantly higher 1 h post-treatment in comparison to controls (0.123 ± 0.03 vs. 0.083 ± 0.011, *p* < 0.05) (Figure 8A). However, gene expression levels of *VEGF* were not different at 4 h and 24 h following treatment between controls and embryos treated with the inhibitor (*p* > 0.05, Figure 8B,C). The relative mRNA expression levels of *VEGF* were also significantly higher at 60 + 1 h in EEM of Y-27632-treated embryos compared with controls (1.159 ± 0.339 vs. 0.673 ± 0.135, *p* < 0.05) (Figure 8D). Gene expression levels in EEM at 4 h and 8 h following treatment were not different between the control and experimental group (*p* > 0.05, Figure 8E,F).

Relative mRNA expression levels of *VEGFR-2* were also significantly higher in Y-27632-treated embryos 1 h post-treatment in comparison to controls (0.692 ± 0.155 vs. 0.458 ± 0.127, *p* < 0.05) (Figure 8G). At 4 h and 24 h following treatment, gene expression levels of *VEGFR-2* were not different between controls and embryos treated with the inhibitor (*p* > 0.05, Figure 8H,I). In EEM of Y-27632-treated embryos, the relative mRNA expression levels of *VEGFR-2* were significantly higher at 60 + 1 h as well compared with controls (0.324 ± 0.109 vs. 0.147 ± 0.026, *p* < 0.05) (Figure 8J). In EEM at 4 h and 8 h following treatment, gene expression levels were not different between the control and experimental group (*p* > 0.05, Figure 8K,L).

### 3.6. Protein Expression of VEGF and VEGFR-2 (ELISA)

Protein expression of VEGF and VEGFR-2 in embryos and membranes was analyzed 8 h and 24 h following treatment at 60 h incubation with vehicle for the control group or 500 µM of Y-27632 for the experimental group.

**Figure 9:**
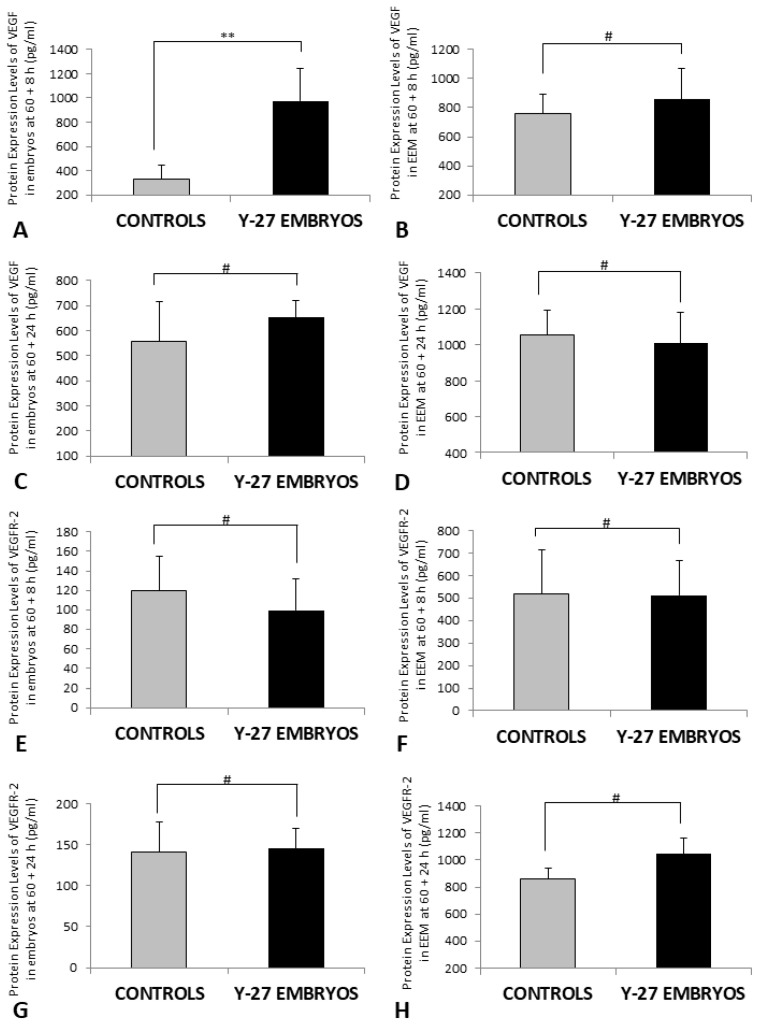
Protein expression of VEGF and VEGFR-2 in embryos (**A**,**C**,**E**,**G**) and EEM (**B**,**D**,**F**,**H**) of the control group and Y-27632 (500 µM) treatment group at 60 + 8 h and 60 + 24 h. Protein expression of VEGF was significantly higher in Y-27632-treated embryos in comparison to controls at 60 + 8 h (**A**, ** *p* = 0.001). Protein levels of VEGF and VEGFR-2 were not different between embryos and membranes of the control and treatment group at other time points (**B**–**H**, # *p* > 0.05). The vertical bars represent the SD. *y-axis*: Protein Expression Levels (pg/mL).

The protein levels of VEGF were significantly higher in embryos in the experimental group when compared with controls at 60 + 8 h (973.0 ± 120.1 vs. 331.0 ± 52.5, *p* < 0.05) (Figure 9A). However, there was no difference between embryos and EEM of controls and Y-27632-treated embryos at other time points (*p* > 0.05, Figure 9B–H).

### 3.7. RT-PCR of Ang-2 and iNOS

Gene expression of Ang-2 and iNOS was analyzed at 60 + 1 h (for Ang-2 and iNOS) and 60 + 8 h (for iNOS) to investigate their contribution in angiogenesis in chick embryos treated with vehicle or 500 µM of ROCK inhibitor Y-27632 at 60 h incubation.

**Figure 10:**
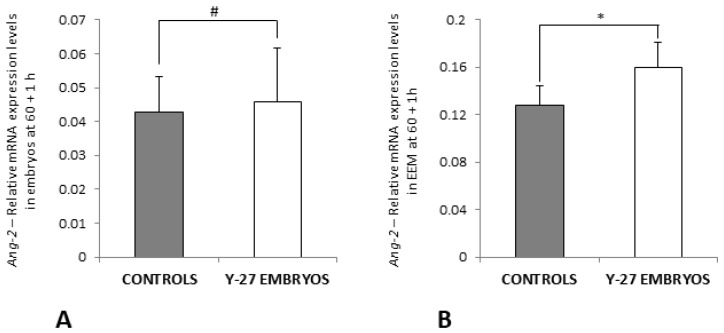
The relative mRNA expression levels of *Ang-2* in embryos (**A**) and EEM (**B**) of the control group and Y-27632 (500 µM) treatment group at 60 + 1 h. The expression levels of *Ang-2* at 60 + 1 h were not different between controls and Y-27632-treated embryos (**A**, # *p* > 0.05) but were significantly higher in membranes of the treatment group when compared to the control group (**B**, * *p* < 0.05). The vertical bars represent the SD. *y-axis*: Relative mRNA Expression Levels.

The relative mRNA expression levels of Ang-2 at 60 + 1 h were not different between controls and embryos in the experimental group (*p* > 0.05, Figure 10A) but were significantly upregulated in EEM of Y-27632-treated embryos at 1 h following treatment in comparison with controls (0.160 ± 0.021 vs. 0.128 ± 0.017, *p* < 0.05) (Figure 10B).

**Figure 11:**
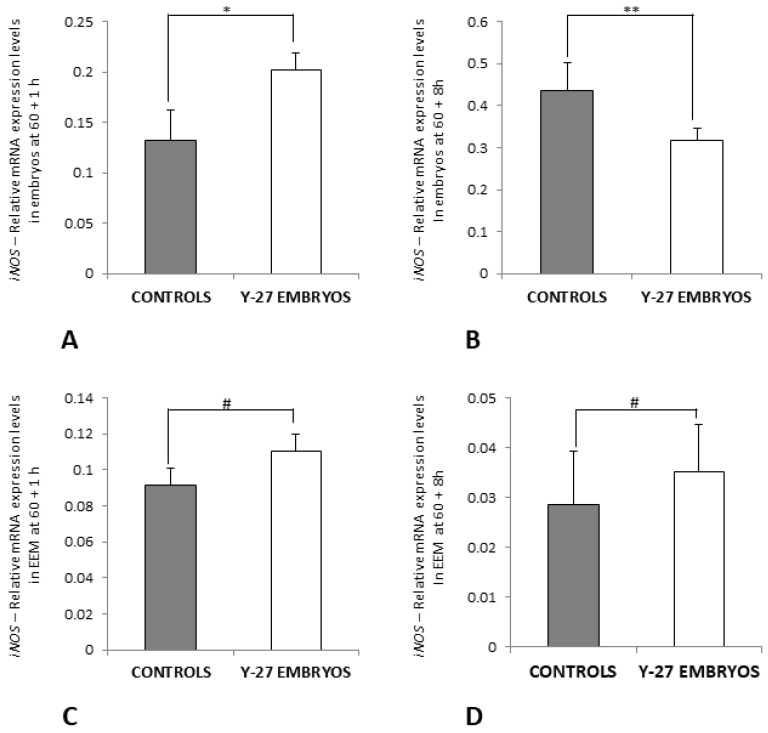
The relative mRNA expression levels of *iNOS* in embryos (**A,B**) and EEM (**C,D**) of the control group and Y-27632 (500 µM) treatment group at 60 + 1 h and 60 + 8 h. The expression levels of *iNOS* at 60 + 1 h were significantly higher in the treatment group when compared to controls (**C**, * *p* < 0.05). The expression levels of *iNOS* at 60 + 8 h were significantly lower in the treatment group when compared to controls (**D**, ** *p* = 0.004). The expression levels of *iNOS* were not different between membranes of the control and treatment group at 60 + 1 h and 60 + 8 h (**C**,**D**, # *p* > 0.05). The vertical bars represent the SD. *y-axis*: Relative mRNA Expression Levels.

Gene expression levels of iNOS were significantly higher in Y-27632-treated embryos compared with controls at 60 + 1 h (0.202 ± 0.056 vs. 0.132 ± 0.075, *p* < 0.05) (Figure 11A), whereas relative mRNA expression of iNOS was significantly lower in embryos in the experimental group in comparison with controls at 60 + 8 h (0.316 ± 0.029 vs. 0.434 ± 0.068, *p* < 0.05) (Figure 11B). The relative mRNA expression levels of iNOS were not different in membranes of the control group and treatment group at 1 h and 8 h following treatment at 60 h incubation (*p* > 0.05, Figure 11C,D).

## 4. Discussion

The major finding of this study is that the exposure of post-gastrulation chick embryos to ROCK inhibitor Y-27632 at 60 h incubation significantly impaired neovascularization, with reduced formation of extra-embryonic blood vessels and reduced circulation of the yolk sac. At very early stages of embryogenesis (60 + 8 h), Y-27632-treated embryos already revealed improper formation of the omphalomesenteric and vitelline vessels in the tail region of the yolk sac compared to controls. Although the formation of extra-embryonic vasculature could be observed in the experimental group throughout development, the process of neovascularization was significantly diminished throughout the period of investigation as confirmed by measurements of ST, the AV, the AOV, and the VI, in addition to fractal analysis at a more advanced developmental stage. Furthermore, the inhibition of ROCK downstream signaling was demonstrated in membranes and embryos when the analysis of the effects on the extra-embryonic angiogenesis and morphology was conducted at 8 h following treatment, as shown by the decreased expression of p-MLC in both tissues. Finally, Y-27632 caused an increased gene expression of VEGF and VEGFR-2 in embryos and membranes at 1 h post-treatment, whereas a significant difference was not seen at later time points. Protein levels of VEGF were higher in Y-27632-treated embryos at 8 h following treatment, whereas no difference was seen in membranes at that time point. Protein expression of VEGF at 60 + 24 h and of VEGFR-2 at 60 + 8 h and 60 + 24 h was not different between embryos and membranes of the control and treatment groups.

Impaired angiogenesis has been connected with multiple pathological conditions in humans, including failure of ventral body wall closure. It has been hypothesized that gastroschisis, one of the most frequently observed VBWDs, results from the premature atrophy or abnormal persistence of the right umbilical vein causing mesenchymal damage to the abdominal wall and intestinal herniation. Alternatively, it has been proposed that the disruption of the right omphalomesenteric artery can lead to the development of gastroschisis [51,52].

VEGF is the crucial regulatory growth factor orchestrating vascular development during embryogenesis (vasculogenesis) as well as blood vessel formation (angiogenesis) in later stages of development [53,54]. During angiogenesis, VEGF is involved in the main processes of neovascularization, mainly by regulation of gene expression and alterations of the actin cytoskeleton [55,56]. The latter, in association with cell–matrix adhesion, are the key functions in endothelial cell migration in angiogenesis [56]. The biological functions of VEGF are exerted upon binding to three receptor tyrosine kinases, known as VEGFR [35,54]. VEGFR-2, the main receptor on endothelial cells, is predominantly described as the leading receptor in angiogenic signaling [35]. Mice deficient in VEGFR-2 have been reported to die in utero at 8.5 days post-coitum from early defects in the development of hematopoietic and endothelial cells. The authors demonstrated the absence of yolk sac blood islands and organized blood vessels as well as reduction in hematopoietic progenitors [57,58]. This phenotype is similar to that of VEGFA^−/−^ mice revealing impaired angiogenesis and blood island formation leading to developmental anomalies [59,60]. Therefore, it has been agreed that VEGFR-2 is the major transducer of the mitogenic, angiogenic, and permeability-enhancing effects of VEGF on endothelial cell differentiation, proliferation, migration, and formation of the vascular tube [32,34,35].

The role of the Rho family of small GTPases as key regulators of angiogenesis has been well established, and it also plays an essential role in transmitting signals downstream of VEGF [41,61,62]. Specifically, Rho proteins appear to be direct downstream targets of VEGF-mediated endothelial cell signaling, leading to activation of RhoA, Rac1, and Cdc42 within several minutes after VEGF stimulation [36,44,45]. Following this rapid increase, Rho proteins return to basal levels within 30 min, which emphasizes their importance during very early stages of VEGF-controlled blood vessel formation [36]. In chick embryos, Uchida et al. have shown that spontaneous angiogenesis in vivo is dependent on RhoA using a chick chorioallantoic membrane (CAM) assay [63]. In particular, the RhoA/ROCK pathway is crucial for the fundamental processes in angiogenesis, inducing capillary permeability, migration, capillary cord formation, and the proliferation of endothelial cells, mainly by its effects on the actin cytoskeleton and cell contractility [23,55]. Due to the fact that this pathway is involved in the cytoskeletal function of the vasculature, it has therefore been suggested that the RhoA/ROCK signaling is also related to vascular diseases. However, studies investigating the function of ROCK during angiogenesis and vascular remodeling using inhibitors such as Y-27632 or H-1152 have shown contradictory and inconclusive results so far [38,64].

VEGF-induced endothelial cell migration has been shown to involve dynamic, coordinate changes in cell adhesion, signal transduction, and cytoskeletal dynamics and organization [30]. Van NieuwAmerongen et al. have studied the effects of VEGF on the endothelial cytoskeleton in human foreskin microvascular and human umbilical vein endothelial cells. Stimulation of cells with VEGF was described to cause an increase in cytoplasmic F-actin staining and stress fiber (SF) formation, accompanied by the loss of peripheral F-actin and an increase in active RhoA and its recruitment to the cell membrane. Treatment with Y-27632 disrupted the endothelial F-actin cytoskeleton and VEGF-induced SF formation. The authors demonstrated that the inhibitor abolished VEGF-enhanced endothelial cell migration and reduced the mean tube length of the capillary-like tubular structure formed in response to VEGF and TNF-α in a dose-dependent manner [56]. Similar results were also demonstrated by Hoang et al. investigating RhoA function during neovascularization in a model of mouse skin angiogenesis using VEGF transfectants. Bryan et al. analyzed the role of RhoA/ROCK signaling in various essential processes during angiogenesis, such as endothelial cell migration, survival, and cell permeability. Using retinal explant cultures from bovine eyes, the authors demonstrated that VEGF strongly promotes vessel outgrowth, which was significantly disrupted by the addition of Y-27632. ROCK inhibition was shown to reduce VEGF-induced permeability, but it did not affect VEGF-mediated proliferation, which is similar to findings presented by Hoang et al. The compound also impaired VEGF-mediated tube morphogenesis, leading to an improper vascular network consisting of flattened multicellular structures [53].

All in all, these aforementioned results obtained from various cell types suggest that ROCK signaling is heavily involved in numerous and fundamental mechanisms during angiogenesis, resulting in immature or reduced blood vessel formation following treatment with agents inhibiting the ROCK pathway, which is in agreement with our findings. Treatment of chick embryos at 60 h incubation with ROCK inhibitor Y-27632 significantly decreased the formation and amount of blood vessels in the extra-embryonic membranes throughout the embryonic development. At 60 + 8 h, the most striking effects on the vasculature could be seen in the experimental group, revealing impaired development of omphalomesenteric and vitelline vessels in the tail region. During the investigation period, vessel formation appeared to proceed but was strongly delayed and decreased compared to the control group, as confirmed by calculating the length of ST and areas of vasculature as wells as fractal analysis [26].

**Figure 12:**
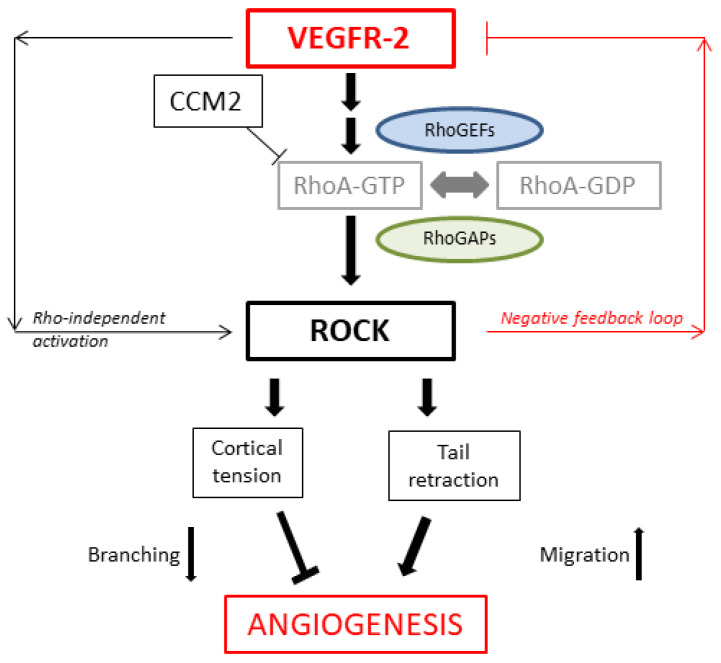
Dual role of ROCK signaling in regulation of angiogenesis; adapted from [38].

VEGF-induced migration of endothelial cells involves actin reorganization. Beside phosphorylation of MLC, VEGF has been stated to regulate the phosphorylation and activity of focal adhesion kinase (FAK) as well, which is important for focal adhesion assembly and disassembly as well as actin organization [35,65]. Hence, VEGF in association with paxillin and talin or vinculin leads to the recruitment of FAK to focal adhesions. VEGFR-2 also transduces its mitogenic functions by influencing FAK, thus regulating cytoskeleton organization and migration [35]. Kroll et al. have recently provided important in vivo evidence for the interaction of VEGF and VEGFR-2 during angiogenesis and have even postulated a potentiating effect of Rho kinase inhibition using H-1152 on VEGF-driven retinal neovascularization and sprouting angiogenesis in human umbilical vein endothelial cells. Pharmacological inhibition as well as expression silencing of ROCK I and ROCK II in endothelial cells activated VEGF-stimulated activation of VEGFR-2, which is necessary to induce VEGF-induced migration, proliferation, NO release, and permeability. The H-1152-driven increase in blood vessel growth has been stated to be completely dependent on VEGFR-2 activation, suggesting a negative feedback loop in which VEGF-induced ROCK I and ROCK II activation counteracts VEGFR-2 activation [64]. Van NieuwAmerongen at et al. have seized on the Kroll conclusion and postulated a dual role for ROCK in the regulation of angiogenesis (Figure 12) [38].

Although we recorded a significant decrease in blood vessel formation following ROCK inhibition in our experiments, which is contrary to the previously mentioned results, we still wanted to investigate the potential effects of Y-27632 on VEGF and VEGFR-2 activity based on Kroll’s assumption [64]. At 60 + 1 h, gene expression levels of VEGF and VEGFR-2 were significantly upregulated in membranes and embryos in the experimental group, which is in line with the negative feedback loop presented by Kroll et al. This may reflect an early inhibitory effect of Y-27632 on ROCK signaling in angiogenesis and subsequently explain the activating effect on VEGFR-2 and VEGF mRNA levels. Y-27632 seems to have a very rapid inhibiting effect on vessel formation during angiogenesis when compared to the effect on morphologic development in the embryos themselves. The ROCK inhibitor may affect neovascularization soon after administration, hence disrupting the underlying processes and mechanism necessary for further vessel formation, which may then lead to the reduced extra-embryonic vascularization at later stages of embryonic development [66,67]. It has been shown that during development, the first hematopoietic cells arise in the yolk sac from mesodermal structures and differentiate into endothelial and blood cells, forming blood islands [26,68]. Therefore, the effects of ROCK inhibitor Y-27632 on the extra-embryonic membranes may also contribute to abnormalities in the morphology of the embryos, as described by the authors in recent studies [20,21,22]. The impact and chronology of this improper supply of nutrients provided by the yolk sac may be highlighted by the early effect of Y-27632 on the extra-embryonic vasculature recorded at 60 + 1 h and the delayed morphological anomalies seen at 60 + 8 h. The mRNA levels of VEGF and VEGFR-2 were not different at 4 h and 8 h in Y-27632-treated membranes and embryos. This may indicate an early compensatory effect in the experimental group. To further examine possible post-translational effects of Y-27632 on VEGF and VEGFR-2 in later stages of development, we studied protein levels in membranes and embryos at 8 and 24 h following the administration of vehicle or the inhibitor. Protein expression of VEGF was only higher in Y-27632-treated embryos at 60 + 8 h, which may reflect a compensatory effect in protein levels following ROCK inhibition. However, no significant differences in protein expression of VEGF and VEGFR-2 were recorded in the remaining ELISA procedures. Hyvelin et al. have found similar results in their study investigating the role of the RhoA/ROCK pathway in the development of chronic hypoxia-induced pulmonary hypertension and the associated structural changes in the pulmonary vasculature. Inhibition of ROCK signaling with Y-27632 abrogated the development of hypoxic pulmonary hypertension by suppressing sustained pulmonary vasoconstriction and hypoxia-induced vascular remodeling revealing improperly formed intra-acinar vessels. Moreover, the compound did not alter the expression of the pro-angiogenic genes VEGF-A and Ang-1 after one week of hypoxia. However, the authors did suggest that the expression of these genes might have been affected by Y-27632 at earlier time points, thus inhibiting angiogenesis [69].

We also tested other genes which might have influenced the impaired vessel formation in treated embryos. Ang-2 is a complex regulator of angiogenesis that plays a central role in both vessel sprouting and vessel regression. It is almost exclusively produced by endothelial cells and functions mainly as a vessel-destabilizing molecule by regulating the vessel maturation and remodeling of Ang-1/Tie2 in an antagonistic manner [70,71,72]. It has been demonstrated in previous studies that Ang-2 prevented the formation of capillary-like structures and impaired angiogenesis in a CAM chick model [71]. However, in contrast, Ang-2 has also been shown to phosphorylate the integrin adaptor protein FAK inducing sprouting angiogenesis in a Tie2-independent manner [70]. Maisonpierre et al. generated transgenic mice overexpressing Ang-2 in their blood vessels and found that the animals were smaller and exhibited an abnormal vascular phenotype. The authors suggested that this factor may play an early role at the sites of vessel invasion [72]. We found an upregulation of Ang-2 at 60 + 1 h in membranes of Y-27632-treated embryos and found that treated embryos were smaller and exhibited abnormalities. The upregulation may contribute to the disruption of vascular formation by blocking the Ang-1/Tie2 signaling axis or may reflect a compensatory effect, as it has been stated that in the presence of abundant VEGF, as seen at 60 + 1 h in our study, Ang-2 can promote vessel sprouting by inhibiting a stabilizing Ang-1 signal [73].

Next, NO is known to play an integral role in the development and maintenance of the microvascular network, including angiogenesis and vascular permeability [38,74,75]. VEGF enhances NO production and promotes eNOS and iNOS expression in endothelial cells in vitro [74]. Inhibition of ROCK, in turn, has been described to induce increased expression and activity of eNOS [38,64]. Fukumura et al. have analyzed the relative contribution of eNOS and iNOS in VEGF-induced angiogenesis and vascular permeability and found that eNOS plays the predominant role in these processes. Nevertheless, the authors have postulated that iNOS selective inhibitor and iNOS gene deletion slowed angiogenesis in VEGF-containing gels, although it was less effective than eNOS gene deletion. Activation of VEGFR-2 has also been shown to lead to an upregulation of iNOS protein in human umbilical vein endothelial cells [76]. The iNOS gene expression was not altered in membranes treated with Y-27632 compared with controls in our analyses, whereas an upregulation of iNOS mRNA levels was noted at 60 + 1 h in treated embryos, which seems to be associated with the noted upregulation of VEGF and VEGFR-2 at that time point. In contrast, gene expression levels of iNOS decreased in the experimental group during development and were significantly different compared with controls at 60 + 8 h, possibly contributing to the delayed angiogenesis as a consequence.

As already mentioned, endothelial cell migration involves cytoskeletal dynamics and organization, mechanisms to which normal ROCK signaling is fundamental. ROCK regulates actomyosin contractility, an essential process in endothelial cell migration, by direct phosphorylation of MLC and phosphorylation and inactivation of myosin phosphatase, thereby contributing to the formation of multicellular, precapillary cord-like structures arranged by endothelial cells [26,53]. The effects of Y-27632 on the cytoskeletal arrangements in chick embryos have recently been shown by the authors to lead to the failure of ventral body wall closure at later stages of development [20]. In the present study, Western blot analysis showed inhibition of ROCK downstream signaling in both membranes and embryonic tissue in the experimental group at 8 h following treatment with Y-27632, with significantly decreased expression of phosphorylated MLC when compared with controls.

Taken together, the major impact of ROCK inhibitor Y-27632 in angiogenesis seems to be on the cell migration of endothelial cells. The impaired regulation of the cytoskeletal rearrangement may affect and delay the process of organization of endothelial cells into capillary structures, subsequently leading to the attenuated vascularization observed in the experimental group. The vascular defect in the yolk sac and regression of pre-existing vessels noted at very early stages during development impede the maintenance of proper circulation, possibly causing developmental delays and morphological abnormalities in Y-27632-treated embryos, all of which were noted in follow-up investigations at more advanced stages of development [21].

## 5. Conclusions

In conclusion, our experiments have shown that exposure to ROCK inhibitor Y-27632 in the critical period of ventral body wall formation in chick embryos strikingly impairs extra-embryonic angiogenesis, leading to delayed and reduced vessel formation in further development. We speculate that Y-27632 also disrupts the rearrangement process of the cytoskeleton required for VEGF/VEGFR-2-induced endothelial cell migration to establish the regular vasculature in membranes and embryos. Hence, defects in vascularization may contribute to morphological abnormalities during early development and subsequently to a deficient formation of the embryonic tissue, which may result in the rupture of the body wall and intestinal herniation through the defect. Further work to determine the contribution of ROCK signaling or its inhibition in angiogenic processes will be subject of future studies.

## Figures and Tables

**Table 1:** Overview of procedures as described in Material and Methods.

Procedure	Time Point
Treatment	at 60 h incubation
Gross Morphology of extra-embryonic vasculature	60 + 8 h, +24 h, +48 h, +4 d
Measurements of EEM	60 + 24 h
Fractal analysis	60 + 4 d
Western Blot	60 + 8 h
Real-Time qRT-PCR	60 + 1 h, +4 h, +8 h/+24 h
ELISA	60 + 8 h, +24 h

**Table 2:** Oligonucleotide primers used for RT-PCR analysis.

Gene	Sequence (5′-3′)
GAPDH	
*Forward*	cctctctggcaaagtccaag
*Reverse*	ggtcacgctcctggaagata
VEGF	
*Forward*	caattgagaccctggtggac
*Reverse*	catcagaggcacacaggatg
VEGFR-2	
*Forward*	gacagtggcatggtgttcag
*Reverse*	gtgcagtttccttcctggag
Ang-2	
*Forward*	ttgaggaggttggacagttc
*Reverse*	gcttcatttccttcccagtc
iNOS	
*Forward*	agtggtatgctctgcctgct
*Reverse*	ccagtcccattcttcttcc

## Data Availability

Not applicable.
